# Nurses' experiences of supporting patients requesting voluntary assisted dying: A qualitative meta‐synthesis

**DOI:** 10.1111/jan.15324

**Published:** 2022-06-24

**Authors:** Margaret Sandham, Melissa Carey, Emma Hedgecock, Rebecca Jarden

**Affiliations:** ^1^ School of Clinical Sciences, Auckland University of Technology Auckland New Zealand; ^2^ School of Nursing, University of Auckland. School of Nursing and Midwifery University of Southern Queensland Toowoomba Australia; ^3^ Oceania Healthcare Tokoroa New Zealand; ^4^ Department of Nursing, Melbourne School of Health Sciences, Faculty of Medicine, Dentistry and Health Sciences The University of Melbourne, Austin Health Heidelberg Australia

**Keywords:** end‐of‐life care, medical assistance in dying, nursing, palliative care, qualitative meta‐synthesis, qualitative studies, systematic review, voluntary assisted dying

## Abstract

**Aim:**

Describe the reported lived experiences of nurses who have participated at any stage of voluntary assisted dying (VAD), from the initial request to the end of life.

**Design:**

A qualitative meta‐synthesis.

**Data sources:**

Databases searched were CINAHL, MEDLINE, Emcare, Scopus and PsycInfo. The search was undertaken in September 2021 with no date limitations. Qualitative studies were considered if published in English, reported primary data analysis of nurses' experiences who had been involved in VAD and reported direct quotes from nurses.

**Review methods:**

Qualitative studies meeting the selection criteria were critically appraised, then an open card‐sort method was applied. Quotes from nurses were organized to group similar experiences, constructing themes and metaphors across studies as a new understanding of nurses' experiences of VAD.

**Results:**

Eight studies were included. Three major themes were constructed: An orderly procedure, reflecting the need for structure to feel adequately prepared; A beautiful death, reflecting the autonomy the patient exercised when choosing VAD facilitated an exceptionally positive death; and Psychological and emotional impact, where nurses recognized the emotional and ethical weight that they carried for themselves and the team when undertaking VAD.

**Conclusion:**

Nurses may benefit from clear policy, supervision and communication training to support them as countries transition to providing VAD services. Policy provides nurses with confidence that they are undertaking the steps of VAD correctly and provides a layer of emotional protection. Communication training specific to VAD is necessary to prepare nurses to recognize their own emotional experiences when responding to the needs of the patient and their family.

**Impact:**

VAD is increasingly becoming a legal option that nurses are encountering in their professional practice. Understanding nurses' experiences of being involved in VAD is required to support nurses in countries where VAD is becoming available to prepare professionally and psychologically.

## 1 INTRODUCTION

Increasingly, medical procedures related to assisting someone who has a life‐limiting illness to end their lives have been legalized (Woodruff, 2019). Nurses have a unique contribution to end‐of‐life care as the health professional who is often at the bedside in those final moments, and is left to answer the questions that the family may have long after the medical team have finished their part of the procedure (Browall et al., 2014). Much of the existing literature and research in voluntary assisted dying (VAD) has been concerned with health professional attitudes (Terkamo‐Moisio et al., 2019) and the medical professional's role (Bélanger et al., 2019). Little is known about the experiences of nurses in caring for individuals and families of people who make this choice. This systematic review and meta‐synthesis aims to analyse previously reported nurses' experiences where VAD is legal, using meta‐ethnography. This new knowledge will inform the preparation of nurses in countries where VAD is soon to be legalized. For example, VAD became available in New Zealand in November 2021 ('End of Life Choice Act', 2019), and although two Australian states have recently legalized VAD, three of the four remaining Australian states have passed legislation to follow in 2022 and 2023 (White & Willmott, 2018).

## 2 BACKGROUND

Palliative care services within New Zealand are presently responding to requests for VAD and some services have taken a position on whether they will offer VAD as a service. Nurses are at the frontline of these requests for VAD. The differentiation between palliative care and services providing VAD has been an ongoing discussion as more countries legalize VAD. Palliative care concerns itself with the holistic care of those towards the end of their life who are experiencing health‐related suffering that cannot be relieved without medical intervention, and compromises physical, spiritual, social and/or emotional functioning. Furthermore, it aims to improve the quality of life of patients, their families and their caregivers (Radbruch et al., 2020). Combining VAD into palliative care services has led to new models of practice and policy at times at odds with the services' foundational philosophy (Freeman et al., 2021). These larger issues around the model of care include bringing to light previous experience of nurses to prepare and know how to support nurses who are navigating new experiences in their professional careers and workplaces, under the gaze of the community (Auret, 2022; Freeman et al., 2021).

Understanding global experiences of nurses in the transition to assisted dying as an end‐of‐life care option will enable implementation of strategies aimed at supporting nurses for who new legislation means considering their role in VAD. Emerging research suggests that VAD may present an existential crisis for nurses who can empathize with the desire for a good death yet are concerned with the ethics of the planned unnatural death (Bellens et al., 2020; Denier et al., 2009). Although nursing practice in VAD improves with experience, the emotional impact does not lessen over time (Denier et al., 2010; Elmore et al., 2018) (Bellens et al., 2020). Indigenous nurses may face the most significant existential crisis as they are often the bridge between vulnerable communities and the health care services. Dying in place is an important consideration for VAD for Indigenous peoples, as the choice to stay on traditional lands (whenua or country) at the end of life and receive effective palliative and end‐of‐life care remains elusive (Cottrell & Duggleby, 2016; Oetzel et al., 2015).

A preliminary search of the literature revealed a prior synthesis describing nurses’ experiences with VAD which reported their objective was to understand the policy, practice and ethical implications of assisted death for nursing (Pesut et al., 2019). The six‐article synthesis had several methodological limitations including that it included two studies that had the same participants, and others that included nursing students, nurse assistants and other health professionals without uniquely identifying nurses. Our study offers a recognized methodology and focuses exclusively on reporting nurses' experiences through the analysis of their quotes.

## 3 THE REVIEW

### 3.1 Aim

We will describe the reported experiences and learnings of nurses who have participated in the process of legal VAD to inform strategies to support nurses in countries where VAD is soon to be legalized. The review question was as follows: ‘What are the experiences of nurses supporting patients' seeking voluntary assisted dying?’

### 3.2 Context

The study authors resided in New Zealand and Australia in the months leading up to the legalization of VAD in New Zealand and parts of Australia.

### 3.3 Terminology

The present article has chosen voluntary assisted dying (VAD) as best representing the phenomena of interest. Suicide is often considered an individual act which has a long history of religious stigma, while medically assisted death is the death with assistance of others within an accepted legal framework. In contrast to the active nature of VAD, palliative sedation is a passive act, mostly concerned with the mitigation of pain and discomfort and the delivery of medication until the person dies. Terms such as voluntary euthanasia, physician‐assisted suicide and voluntary assisted dying are often used interchangeably. For an overview of terminology, see Death with Dignity (2021).

### 3.4 Positionality

The authors have taken the stance of information seeking and understanding the experiences of nurses rather than being for or against VAD. We are nurse academics and a Palliative Care Nurse Practitioner from Australia and New Zealand of Māori and New Zealand European descent. The researchers shared the experience of having nursed people at the end of their life and using palliative sedation, but none of us had experience with VAD.

### 3.5 Design

The systematic literature search and quality appraisal drew from the Joanna Briggs Institute (JBI) qualitative systematic review methodology (Aromataris & Munn, 2017), the seven‐step meta‐ethnography (Malterud, 2019; Noblit & Hare, 1988), and were reported according to the framework of enhancing transparency in reporting the synthesis of qualitative research (ENTREQ; Tong et al., 2012).

### 3.6 Search methods

#### 3.6.1 Registration

PROSPERO CRD42021276819.

#### 3.6.2 Search strategy (step 1)

We sought primary research studies. First, we ran a preliminary search of the Cumulative Index of Nursing and Allied Health Literature (CINAHL) and Medical Literature Analysis and Retrieval System Online (MEDLINE) to identify relevant terminology and keywords to include in the search strategy (phase 1). The text words contained in the titles, abstracts and keywords were used to develop the full search strategy for all databases. Phase 2 adapted and implemented the full strategy. Finally, the reference lists of articles selected for data extraction were searched for additional studies.

#### 3.6.3 Inclusion/exclusion criteria

##### Population

Included were Registered Nurses, Enrolled Nurses, Nurse Practitioners, or international equivalent terms for nurses who have a license to practice nursing. We included nurses of all ages, cultures, ethnicities, genders and experience levels. We excluded studies where veterinary nurses involved in animal euthanasia were the population, and studies where nurses were not uniquely identified as a separate group. Student nurses and nursing assistants were also excluded.

##### Phenomena of interest

Studies were included if they reported the experiences of nurses who had observed first hand, been on a team where VAD had been requested or had a patient request VAD from them, or otherwise participated in the VAD process. Studies were excluded if they reported illegal euthanasia, palliative sedation or explored attitudes and beliefs of nurses who had not participated in VAD.

##### Context

We considered all settings where VAD was legally practiced and all geographical areas. These settings included but were not limited to at home care, aged residential care, hospital and private clinics. We included all human patient populations where nurses may encounter requests for VAD, which may include paediatric, adult, older adult and mental health.

##### Study designs

Types of publications included peer‐reviewed journal articles. Types of studies included primary qualitative research studies reporting quotes from nurses caring for humans at the end of life. All age ranges of studies and qualitative methodologies were included. The studies must have been presented in English or had a translation available. The characteristics of primary studies are presented in Table 1.

##### Information sources

Databases searched were CINAHL, MEDLINE, Emcare, Scopus and PsycInfo.

##### Study selection

All records identified from the search were uploaded into EndNote (X9; Clarivate) and duplicates removed. Four independent reviewers (MS, RJ, ES and MC) screened the title and abstract of potentially eligible studies against the inclusion and exclusion criteria for the review. Full text of potentially relevant papers was retrieved and their citation details were imported into the Covidence systematic review software. The full texts of eligible citations were screened in detail against the inclusion criteria by all reviewers. Reasons for exclusion were reported. Disagreements arising between the reviewers at any stage were resolved through discussion. Search results are presented in a Preferred Reporting Items for Systematic Reviews and Meta‐Analyses (PRISMA) flow diagram (Page et al., 2021).

#### 3.6.4 Quality appraisal (step 2)

Studies that were shortlisted for inclusion were assessed by RJ and MS using the JBI. Reviewer conflicts were resolved through discussion. Results are reported as a narrative summary table with dependability scoring according to Munn et al. (2014). All studies were put forward for synthesis irrespective of quality due to the various ontological and epistemological stances on systematic reviews.

### 3.7 Data abstraction

Reading and localizing the studies (step 3).

The final included studies were read by two researchers to gain an overview of themes and the experiences of nurses reported.

### 3.8 Data synthesis

#### 3.8.1 Determining the relationships between studies (step 4)

A stepwise approach informed by Noblit and Hare (1988), Malterud (2019) and Sattar et al. (2021) was used to guide extraction of the themes and metaphors of nurses' experience to Microsoft Excel™. As a starting point, we identified a rich index study. When studies contained nurses and other health professionals' quotes, the themes of the authors, alongside nurses' quotes, and were put forward for analysis in this study.

#### 3.8.2 Translating studies into one another (step 5) and synthesizing translations (step 6)

In the context of the themes and metaphors identified in step 4, quotations from nurses were explored by two researchers (RJ and MS) independently using a pre‐design open‐sort technique (Paul, 2008) (Rugg & McGeorge, 1997). To maintain an alignment between the primary studies’ themes and to ensure that nursing voices were represented, we included these alongside themes and metaphors in a matrix. After our initial reading and independent analysis, the team then met and discussed excerpts of primary studies taking an iterative interpretive approach. We considered how the content of the matrix might be interpreted, facilitating the analysis to be expressed as new themes. Quotes from the nurses were organized into similar themes and experiences constructing themes and metaphors across studies as a new understanding of nurses' experiences of VAD. Cross checking of the final synthesis was undertaken by two additional researchers (authors MC and EH) by reviewing the quotes in categories and names of the themes.

## 4 RESULTS

### 4.1 Search outcome (step 1)

**FIGURE 1:**
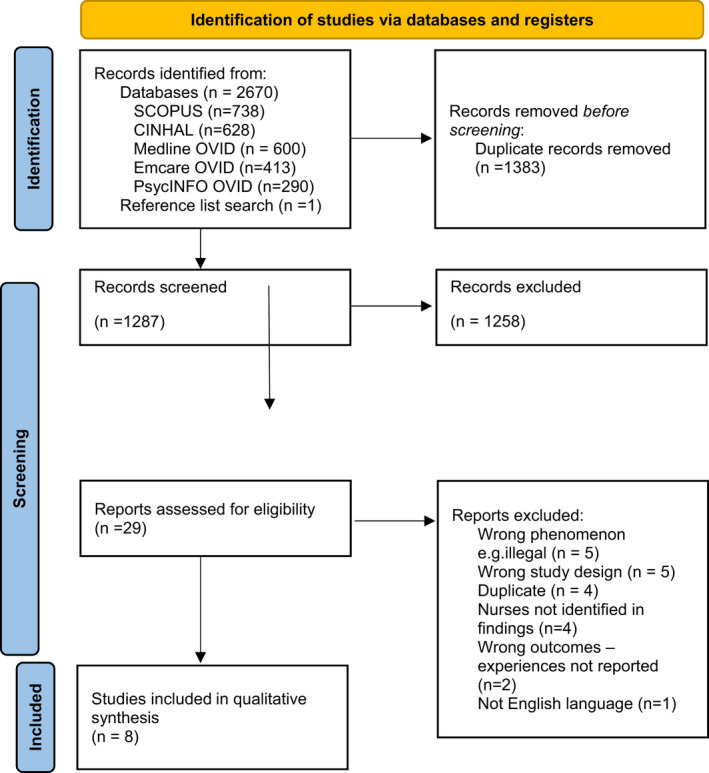
Flow chart of search process.

The study search took place in September 2021. Database searches yielded 2670 articles, and one additional article was sourced from included studies’ reference lists. Duplicates were removed and 1289 articles remained, 29 full texts were screened and of those 8 met the selection criteria. Figure 1 shows a flow chart of the search screening process. The majority of studies that were excluded during the title and abstract screening were due to the population or study design being outside of the selection criteria. Studies excluded during full‐text screening were mainly due to nurses not being identifiable in the population, the wrong phenomenon (e.g., describing practices in countries where euthanasia was illegal) or the wrong design (no nurses' quotes included).

### 4.2 Study characteristics

Methodological approaches of the eight included studies were heterogeneous and included narrative (*n* = 3), grounded theory (*n* = 2), interpretive description (*n* = 1), qualitative descriptive (*n* = 1) and other (*n* = 1). Most studies drew from thematic analysis to construct themes. Studies were set in Canada (*n* = 5) and Belgium (*n* = 3). Study characteristics are reported in Table 1. Sample sizes of nurses ranged from 1 to 43 and represented the empirical data from 168 nurses from Belgium and Canada who worked in a range of hospital, hospice, community, private clinics and palliative care settings. Ages, experience level, culture and gender of nurses were not consistently reported; however, several nurse practitioners were included. No studies uniquely identified Indigenous nurses' experiences.

**TABLE 1:** Characteristics of the primary studies included in the analysis

Author (citation year)	Title	Phenomenon of interest	Country	Context	Methodology / theoretical framework (data collection)	Participants
Bellens et al. (2020)	‘It is still intense and not unambiguous’. Nurses' experiences in the euthanasia care process 15 years after legalization	Nurses' experiences	Belgium	Hospitals (*n* = 20) or in home care (*n* = 8); geographically spread over Flanders	Grounded theory / semi‐structured in‐depth interviews	Nurses (*n* = 28), of which were employed in: hospital (*n* = 11), 8 home care (*n* = 8), bedside nurses (*n* = 16). Nurses were involved in the care of patients requesting euthanasia at least 5 times (*n* = 17), having had experience with euthanasia in the last 6 months (*n* = 16)
Beuthin et al. (2018)	Medical assistance in dying (MAiD): Canadian nurses' experiences	Nurses' experiences	Canada	Urban and rural areas across Vancouver Island, British Columbia, working across settings including acute care, residential care, primary care clinics and community and palliative care	Narrative inquiry and thematic analysis / semi‐structured interviews conducted in person or by phone	Registered nurses (*n* = 15), Nurse Practitioner (*n* = 1) and Licensed Practical Nurse (*n* = 1)
Beuthin (2018)	Cultivating compassion: The practice experience of a Medical Assistance in Dying coordinator in Canada	Reflections on personal experience leading a team conducting MAiD	Canada	MAiD coordinating facility	Narrative (auto)ethnography	Coordinating nurse (*n* = 1)
Bruce and Beuthin (2020)	Medically Assisted Dying in Canada: ‘Beautiful Death’ is transforming nurses' experiences of suffering	Nurses' experiences of suffering	Canada	Diverse settings including acute care, community–home care and specialty areas including emergency room and palliative care	Narrative inquiry / semi‐structured interviews	Registered nurses (*n* = 15), Nurse Practitioner (*n* = 1) and Licensed Practical Nurse (*n* = 1)
Lemiengre et al. (2010)	Impact of written ethics policy on euthanasia from the perspective of physicians and nurses: A multiple case study in hospitals	Impact of policy on euthanasia	Belgium	Participants were from hospitals (*n* = 3) selected based on the (1) availability of an ethical infrastructure, (2) development of a euthanasia policy, (3) format and content of the policy and (4) communication and accessibility of the policy	Grounded theory / in‐depth interviews	Physicians (*n* = 11, excluded) and palliative care specialist nurses (*n* = 12)
	Impact of Medical Assistance in Dying on palliative care: A qualitative study	Experiences of palliative care providers	Canada	Inpatient consult services, inpatient palliative care units, outpatient clinics, home‐based palliative care and residential hospices. Several participants worked in more than one setting	Qualitative descriptive using thematic analysis / semi‐structured interviews	23 Palliative care providers from which nurses (*n* = 10) were identified
Pesut et al. (2020)	The rocks and hard places of MAiD: a qualitative study of nursing practice in the context of legislated assisted death	Nurses' experiences and practice	Canada	Home and community (*n* = 32; 54%), acute care (*n* = 10; 17%), LTC (*n* = 5; 9%), hospice (*n* = 4; 7%), clinic (*n* = 3; 5%) and other (*n* = 5; 9%)	Qualitative using interpretive description / semi‐structured interviews	Registered Nurse (*n* = 43; 73%), Nurse Practitioner (*n* = 13; 22%), Clinical Nurse Specialist (*n* = 3; 5%)
Rys et al. (2015)	Bridging the gap between continuous sedation until death and physician‐assisted death: A focus group study in nursing homes in Flanders, Belgium	Nurses' perceptions	Belgium	Nursing homes	Not reported / focus group using semi‐structured interviews	48 clinicians, from whom nurses (*n* = 24) were identified (Coordinating nurses *n* = 4, head nurses *n* = 7 and nurses *n* = 13)

### 4.3 Quality appraisal (step 2)

Most studies were of high quality and met many of the JBI quality criteria, but most did not locate the researchers theoretically, politically, include a statement on their stance around VAD or engage in reflexivity (Table 2). One study showed a predominance of quotes from senior clinicians despite having a broad participant group.

**TABLE 2:** Quality appraisal of studies

Author (citation year)	Qu.1	Qu.2	Qu.3	Qu.4	Qu.5	Qu.6	Qu.7	Qu.8	Qu.9	Qu.10	Dependability (score from sum of yes votes for q 2,3,4,6,7) Munn et al., (2014)
Bellens et al. (2020)	Yes	Yes	Yes	Yes	Yes	No	Yes	Yes	Yes	Yes	High
Beuthin et al. (2018)	Yes	Yes	Yes	Yes	Yes	No	Yes	Unclear	Yes	Yes	High
Beuthin (2018)	Yes	Yes	Yes	Yes	Yes	Yes	Yes	Yes	Yes	Yes	High
Bruce and Beuthin (2020)	Yes	Yes	Yes	Yes	Yes	No	Yes	Unclear	Yes	Yes	High
Lemiengre et al. (2010)	Yes	Yes	Yes	Yes	Yes	No	Yes	Yes	Yes	Yes	High
	Yes	Yes	Yes	Yes	Yes	No	Yes	No	Yes	Yes	High
Pesut et al. (2020)	Yes	Yes	Yes	Yes	Yes	No	No	No	Yes	Yes	Moderate
Rys et al (2015)	Yes	Yes	Yes	Yes	Yes	No	No	No	Yes	Yes	Moderate

### 4.4 Data abstraction

#### 4.4.1 Reading and locating the studies (step 3) and determining the relationships between studies (step 4)

The main authors' themes highlighted the complex emotional experiences of the nurses, the qualities of the death observed, the right to choice, organizational structures and policy to support nurses (see Table 3). There was heterogeneity in the methods and participants, but an overrepresentation of nurses from Canada and Belgium, with one author present in the majority of Canadian publications. Two Canadian studies had identical participant characteristics and authors but were not declared to be the same sample.

**TABLE 3:** Themes identified from the primary studies included in the analysis

Author (citation year)	Theme	Description of theme
Bellens et al. (2020)	Intense and not unambiguous	Overwhelming and mixed emotions were experienced by participants involved in MAiD
	Professional fulfilment	Care to MAiD patients gave them a feeling of professional fulfilment
	Frustration	Nurses felt negative emotions often related to feeling that they were not contributing to good patient care
Beuthin (2018)	Holistic care without judgement	‘Participants described the profession as providing holistic nursing care and MAiD as an expression of this care’. p. 513
	Advocating choice	Providing non‐judgemental care and advocacy for the option of MAiD
	Supporting a good death	The nursing role providing comfort, reducing suffering and expanding the possible options of a good death
	Being pioneers	‘Many nurses were aware of the historic role they were playing’. p. 514
	Strongly opposed	Uncertainty, confusion, fear, lack of policy and concern about legal and ethical messages
	In between	Relief that there were more options and feeling positively impacted by MAiD
	Strongly supportive	‘Nurses also described a range of emotions–‐some anticipated, and others not. Stories of “being emotional” included feeling choked up or shedding a tear’. p. 517
	Nursing practice	Nurses required a specific set of communication and practical skills to engage in MAiD
	Technical skills	Being able to establish intravenous access
	Communication	‘The combination of having excellent technical capacity with requisite communication skills was described as essential’. p. 518
Beuthin (2018)	The calling	‘… my coming to this work felt like “a calling.” From a source beyond, an embodied feeling that being in service with MAiD was bringing all in my world, past, present, and future, into alignment’. p. 1683
	Embodiment: Becoming the Face of MAiD	‘I quickly came to be seen as the MAiD person. People told me things they might not share with others, a personal experience with death that had been difficult or beautiful’. p.1684
	Immersion in the Clinical Practice of MAiD	‘This meant accelerated learning, experience, and need for support. I was in the privileged position of witnessing what clinicians were experiencing and learning alongside’. p 1686
	Interactions With Those Seeking MAiD	‘Contacts made came to me directly from individuals with illness or family members, and ensuing conversations were raw, frank and honest’. p. 1686
	Self‐Survival: Sense Making	‘…I heard stories every day, ripe with emotions of suffering or expressions of gratitude that landed on me and I let linger, sink in. I was bearing witness (Naef, 2006) to a unique time in history and to one of the most profound moments in a life, that being death’. p 1687
Bruce and Beuthin (2020)	Prior to MAiD: A culture of nurses' taken‐for‐granted suffering–feeling terrible	‘Nurses shared stories of their own suffering when witnessing patients' pain—their own suffering was often invisible and taken for granted as part of a nurse's role’. p.271
	Prior to MAiD: Witnessing painful deaths	‘Participants encountered patients living painful deaths across a variety of settings’. p. 271
	Prior to MAiD: Patients asking to end their suffering	‘Nurses shared feelings of helplessness and sorrow when patients would hold tightly to their arm asking for medications to hasten death and end their suffering’. p.272
	Prior to MAiD: Providing comfort and (un)intended death	‘Nurses also talked about the ambiguity and at times uneasy practices of administering medications to relieve patients' pain that resulted in expected yet (un)intended death’. p. 272
	Nursing post MAiD: Transformational feelings of a beautiful death	‘…positive experiences expressed in terms such as “beautiful,” “rewarding,” and “amazing.” Nurses shared how MAiD is changing their view of dying overall’. p. 272
	Nursing post MAiD: Beholding peaceful dying	‘“it's very, very peaceful” and “it's quick”’. p.272
	Nursing post MAiD: Gratitude	‘In lieu of suffering, nurses identified gratitude as a primary emotion both from the nature of the work and the appreciative feedback from patients and’ families’. p. 273
	Nursing post MAiD: Residual discomfort	‘…addresses nurses' uncomfortable feelings evoked by unresolved questions and concerns. Two substory lines include worries of becoming desensitized and ongoing deeper questioning’. p. 273
Lemiengre et al. (2010)	Euthanasia policy as a practice manual	The policy being a system to help navigate the process of MAiD
	Euthanasia policy as guideline for Professional Practice	Policy supporting professionals to provide optimum care in MAiD
	Competence	Policy providing the necessary information to inform patients and conduct their role effectively.
	Carefulness	Policy providing a checklist to ensure care is correct and organized, and that the team is supported [check]
	Euthanasia policy: A support for Ethical Practice	Support for ethical practice/ supports the personal stance of practitioner
	Being safe	Policy enables practitioners to check if they fulfilled legal care criteria and protected against prosecution
	Being certain	Being confident about how to act professionally during the euthanasia process
	Increasing openness to euthanasia request	‘…atmosphere was more open towards euthanasia as a result of the euthanasia policy. This openness made it easier for care providers to listen to the patient's euthanasia request and to communicate more clearly and professionally with the patient about his or her request’. p.56
	Increasing willingness to take on responsibility in the euthanasia care process	Nurses' attitudes and openness towards euthanasia progressed when hospital policies were available to guide them
	‘Guiding Person’ as a mediator of the euthanasia policy	The presence of a person who could interpret the policy for clinicians who were providing euthanasia bridging the theory and the practice.
	MAiD offers an alternative dying experience to natural death	Mixed reflections on whether MAiD positively or negatively impacted the experiences surrounding death and the death itself, but recognition of it differing from natural death
	The laws around MAiD may pose challenges to traditional symptom control strategies	‘…conflict between maintaining Medical Assistance in Dying eligibility and effective symptom control. One of the ways this conflict manifested was in withholding symptom control medications that could cause sedation or confusion and could jeopardize eligibility, as patients needed to be capable of consent at the time’. p. 450
	MAiD creates new ‘difficult conversations’	Practitioners found it difficult to have conversations around MAiD including ethical and moral dilemmas that the process raised in respect to their patients.
	MAiD had an emotional and personal impact on palliative care providers	Emotional and personal toll on providers of palliative care as they navigated the ethics of MAiD
	MAiD changes the patient palliative care provider relationship	‘patients' thought that palliative care included assisted death, which complicated their relationships with these patients’. p. 451
	Palliative care resources are consumed by MAiD requests	Heightened awareness of MAiD seen raising the profile of palliative care, however, diverting resources from palliative care to MAiD
Pesut et al. (2020)	Systems: influential leaders setting the tone	Organizational policies with respect to MAiD existed on a spectrum from none to highly organized, reflecting the organizational leadership had chosen to approach MAiD
	Teamwork: Two's a team	‘Even as they found themselves with varying degrees of team support, participants described teamwork as essential to a successful MAiD process’ p. 6
	Processes: patient‐centred aspirations in a complex system	MAiD deaths as complex and a desire to engage in a professional, organized and patient‐centred process
Rys et al. (2015)	Unconsciousness	The state of unconsciousness as differentiating between MAiD deaths and patients who receive continuous sedation until death
	Pace of the dying process	‘Practically, all nursing home clinicians consider the pace of the dying process an important element for distinguishing CSD from PAD since PAD typically causes immediate death while CSD results in a more gradual process of dying’ p.413
	Emotional burden	‘When comparing CSD with PAD, most clinicians mentioned the differences in the degree of emotional burden. Several clinicians find CSD emotionally easier to perform’. p. 413

## 5 SYNTHESIS

### 5.1 Translating studies into one another (step 5) and synthesizing translations (step 6)

Beuthin et al. (2018) was chosen as the rich index study and was assessed as high quality (Table 2). The nurses' voices were explored and grouped together into three themes, each with three sub‐themes that strongly represented the voices.

**Theme 1. An orderly procedure** *‘to make sure…. that everything is there’* (Lemiengre et al., 2010, p. 54).

Nurses involved in VAD strongly recognized the need for structures to support them in an area that had high emotional and professional risks associated. They lent on traditional structures of policy, being prepared and having the right knowledge, as well as drawing from the knowledge of their team. They reported the necessity of an orderly procedure that was well resourced to enable VAD to run smoothly.

**Sub‐theme 1.1. Creating a shield through preparation** *‘…otherwise, we will loosen the ground under our feet…’* (Lemiengre et al., 2010, p. 54). Nurses heavily emphasized their vigilance around accurately following correct policy and procedures when available. These policies were a safety net for nurses working in an area where they felt that there was a high potential for legal or ethical missteps. Following procedures and in a way, using the technical aspects as a shield, allowed nurses to engage with the patient in a way that they felt was calm and competent, reassuring themselves alongside the patient.

**Sub‐theme 1.2. Interdependency within the team for a smooth VAD process** *‘…we all need to help each other…’* P3 (Pesut et al., 2020, p. 9). Nurses valuing of teamwork ranged from the acknowledgment that they were a team member to experiencing emotional and practical support from discussions with colleagues involved in VAD. Meaningful interactions offering support to a colleague through touch or physical presence were spoken of. In contrast, newer team relationships created a barrier for nurses to advocate for their patients, raising complex emotions. There were very limited discussions of debriefing after a death, and no formalized debriefing was evident.

**Sub‐theme 1.3. VAD being resource intensive and requiring an additional level of planning** *‘…so that everything lines up…’* P25 (Pesut et al., 2020, p. 9). Nurses articulated the practicalities of establishing IV access and ensuring that all the technical checklists of VAD were met. Nurses spoke of VAD being resource intensive and time pressured in contrast to their previous practice. Some of the time pressure related to gaining consent while the patient was competent, whereas in other respects the need to gather all resources and deliver VAD in the necessary or requested timeframe. Some nurses expressed resentment of resources from traditional palliative care services being diverted into VAD.

**Theme 2. A beautiful death** *‘…to help somebody die in the best way possible.’* (Bruce & Beuthin, 2020, p. 273). Nurses involved in VAD reflected on their past experiences with continuous sedation to death and other legal practices prior to becoming involved in euthanasia. They expressed discomfort with some of the deaths they had observed prior to VAD where they perceived that the patient had suffered, or the experience was uncomfortable for those involved. They contrasted the experience of VAD where the clinical team, family and patients were connected and working together to enable the patients to have the best possible experience through control over their environment, the presence of family members and it being a fast and comfortable death.

**Sub‐theme 2.1. Influenced by past experiences of people suffering in death** *‘…when people were very much finished [ready to die]’ and ‘It's hard to watch, especially when they're struggling.’* (Bruce & Beuthin, 2020, p. 271). Most nurses had prior experience of providing end‐of‐life care to patients outside of the VAD framework. Nurses' prior experiences of non‐VAD deaths were mixed and while some spoke of symptom control and the judicious use of palliative sedation, these were often accompanied by discussing times that despite their best efforts the death not going smoothly, with residual pain, difficulty breathing and perceiving that the patient was ‘suffering’. The time when the patient was in a space where they were sedated and awaiting death was experienced by some nurses as ‘horrific’, ‘creepy’ and they felt helpless, powerless and that the death was at odds with what the patient and family would want.

**Sub‐theme 2.2. Appearing neutral while discussing the option of VAD** *‘no matter what they chose, they had those options on the table and that they could feel supported through the whole process if that's what they chose.’* P12 (Pesut et al., 2020, p. 7). Nurses had a strong sense of their role being to advocate for the patient's rights to choose and to provide information in a neutral and non‐judgemental way. Some nurses reflected that the discomfort from other nurses around VAD compelled them to become a stronger advocate to support patients' autonomy and provide information. A minority of nurses who were conscientious objectors felt unsure of what to do when approached for VAD and if it was acceptable to opt out being part of the process.

**Sub‐theme 2.3. Facilitating a meaningful patient and family experience** *‘…he was sure and he got the death he wanted. That's good.’* (Beuthin et al., 2018, p. 514). This category represented the most nurses' voices. Nurses cautiously described feeling happy and that it was rewarding to help the patient to die in a way that they had chosen, with dignity, peacefully and for the most part in the environment and with who they chose. There appeared to be a unique relationship that the nurse had with the family where they were recognized as being brave and providing something that would be professionally and emotionally challenging. Consequently, there were quotes that spoke of families expressing gratitude for the service that the nurse had provided. Nurses spoke of deaths being ‘beautiful’ and feeling an ‘honour’ and a ‘privilege’ to be able to observe and participate in a personal family experience where emotional and peaceful goodbyes are said.

**Theme 3. Psychological and emotional impact** *‘I think anybody would feel the emotion…’* (Beuthin et al., 2018, p. 517). Nurses expressed a complex range of emotions. Some reflected an existential awareness and their role in a sacred space and time in another's life. Nurses were aware that they were engaging in a momentous, existential moment in the patient's and their family history, in the context of a practice, although legal, has been, and still is, ethically and morally contentious. Nurses were navigating their own emotions alongside supporting team members who were visibly moved or shaken by their experience that were at times subtly acknowledged, but not always openly spoken of.

*‘The physician's hands were shaking, and by all the means, mine were too when I went to put in the intravenous. So as he went to connect… he couldn't connect the syringe to the cap on the end of the intravenous. So without even thinking twice, I put my hand on his shoulder, “take a deep breath*.” *And he did ‐ in, out, and then he was ok'*. (Beuthin et al., 2018, p. 515).

**Sub‐theme 3.1. Crossing a line** *‘…We really had no idea what we were doing…’* P42 (Pesut et al., 2020, p. 4). Nurses expressed an awareness of the magnitude of engaging in VAD and felt ethical discomfort about the act of ending a patient's life. Nurses also were fearful of legal repercussions if they did not follow the protocols or checklists exactly so sought reassurance from colleagues. Words such as ‘killing’ and ‘murder’ were brought up and nurses felt as though they were treading a fine line at times. Nurses were concerned about what could happen if they became desensitized to the gravity of what they were doing.

**Sub‐theme 3.2. Carrying an emotional load of self and others** '*I feel we share a sacred space at this moment, and I am moved, feel the profound weight of it all*' (Beuthin, 2018, p. 1685). Nurses described the intensity and weightiness of engaging in discussions about VAD, the care of the patient and support of family. They also recognized the support role they had for team members’ experiences. The empathy that the nurses expressed had an impact on the nurse, although this was mostly seen as a privilege to share that emotional space with the patient. A minority of nurses reported feeling emotionally drained and somewhat resentful of patients engaging in VAD. Some nurses recognized using tasks and procedures to avoid the difficult emotions that the experience triggered.

**Sub‐theme 3.3. Spiritual experience** ‘*It felt reckless from my rational sense, and yet totally right in a spiritual sense, as if answering a calling. I shivered*.’ (Beuthin, 2018, p. 1683). Nurses were emotionally and spiritually moved by their involvement in VAD and shared that the emotional impact lasted for sometimes weeks after the death. There was a recognition of it being a profound experience that they were able to observe and the emotions that it raised, but at the same time feeling both internal and external expectation that they fulfilled a professional role they were in and should emotionally move on. Nurses spoke of the experience enriching them as a person and giving them a positive emotional experience.

## 6 DISCUSSION

### 6.1 Expressing the synthesis' theoretical underpinnings and discussion (step 7)

We sought to describe the lived experiences reported by nurses who have participated in the care of patients seeking VAD in countries where it was legal. The synthesis reported three themes: (1) An orderly procedure: considering the policy and resourcing to enable VAD to occur smoothly; (2) A beautiful death: where nurses felt they were able to offer a positive death experience through VAD that differed from their past experiences; and (3) Psychological and emotional impact: the heaviness and emotional complexity of navigating VAD alone and together with others. Nurses' experiences were centred on providing the best patient care they could while navigating new professional and personal experiences that were emotionally and ethically weighty. They experienced reciprocity from families who expressed gratitude for supporting the patient to a peaceful and meaningful death.

Although palliative sedation and VAD have different goals, nurses in this study compared and contrasted their participation in these procedures. Nurses described experiences with palliative sedation and other deaths where they perceived the patient had not died well, with perceived benefits of giving choices, control and support for patients seeking VAD. Nurses perceived patients' having access to VAD potentially ameliorated some of their distressing experiences which nurses associated with traditional options.

Nurses' articulated the complex emotions that came from engaging in VAD as well as their role in supporting team members who were experiencing the weight of their participation. Although Beuthin (2018) highlighted the importance of reflexivity in working with VAD, the nurses in our synthesis did not articulate engaging in deeper levels of reflection. This may be a publication bias, as it was noted in our quality appraisal that author reflexivity was a limitation of most studies. However, nurses’ lack of critical reflection and the emphasis on reporting professional fulfilment may be a coping strategy when experiencing complex emotions (Bellens et al., 2020) felt that VAD relieved nurses of the existential suffering experienced when using palliative sedation. Taken together, it appears that while it is true that nurses' experiences of observing death where VAD has been used was overall positive, there may be an element of avoiding the complex ethical and emotional processing that occurs through critical reflection. Although some nurses reported debriefing within a team, there did not appear to be a normalized professional supervision or peer support process after VAD.

## 7 PRACTICE AND POLICY IMPLICATION

### 7.1 Supervision

Although nurses in the present study did not raise professional supervision, receiving supervision may support nurses to develop reflexivity when navigating complex ethical and emotional areas of work such as palliative care (Francis & Bulman, 2019; Keane et al., 2020). The nurses' voices in our synthesis strongly articulated the complexity and emotional burden that they carried alongside learning and undertaking the unfamiliar and weighty practice of VAD. Similar to elsewhere (Pesut et al., 2019), nurses in this synthesis touched on ethical issues of patient autonomy and expressed some moral discomfort with VAD. Interdisciplinary group supervision may have additional benefits of appropriately resolving team conflict that may naturally arise from the emotive nature of VAD as well as provide a safe space for debriefing away from the public gaze.

### 7.2 Communication training

Nurses were at times unprepared for the heavy conversations that came from receiving patients’ requests for VAD. This aligns with previously reported experiences of nurses who had difficulty raising and responding to emotional experiences in palliative care patients (M. H. Sandham, E. Hedgecock, M. Hocaoglu, C. Palmer, R. Jarden, A. Narayanan, & R. J. Siegert, under review). Effective professional communication in end‐of‐life care requires emotional self‐awareness and empathy, alongside the ability to articulate emotional and practical information (Shimoinaba et al., 2015). Furthermore, the goals of communication in VAD may be to ascertain the intent of VAD, achieving peace and prepare for the technical process (Pesut et al., 2019). While policy was helpful for nurses to know *what* to do, they had less confidence when it came to *how* to engage in the conversations. Role playing these conversations appears to particularly develop confidence (Brighton et al., 2018). Interprofessional communication training may also facilitate supportive communication within teams engaged in VAD.

### 7.3 Policy

Nurses valued clear policy to keep them legally, ethically and procedurally safe. They also appeared to use policy to keep emotionally safe when they were overwhelmed by the magnitude of what they were undertaking. Policy should be provided from nursing governing bodies as well as by the workplace and framed in a way that is supportive of nurses rather than fear driven, as outlined in this quote: ‘*I think [regulatory college] planted in my head that depending on what I said, I could put myself in some sort of legal or professional problem’* (Beuthin, 2018 p. 516.) Such an approach could unnecessarily discourage willing nurses from being involved in VAD.

### 7.4 Closing

Nurses' responses to patient deaths in their workplaces vary, however, marking the passing of a patient signals the end of the therapeutic relationship and care provision and leaving the emotion attached to it at work. A nurse in this study described an informal ritual, *‘… And then we had a tea party after… the palliative care director brought in goodies and our clerk made us tea and we invited the son and daughter in and the doctor and the nurse that was in attendance…*. (Jill)’ (Beuthin et al., 2018, p. 518). Ending the therapeutic relationship with the family allowed for an expression of gratitude towards the nurse that was valued by the nurse and affirmed their role. Taking part in debriefing at work also supported the nurses to connect with others who had insider experiences of VAD and protected them from the uncertain gaze of the outside community.

### 7.5 Strengths and limitations

The strength of combining nurses' voices to amplify their messages also brings the drawback of removing them from their context and concentrating the methodological weaknesses of previous studies. There may be a publication bias in articles we included. The Western, educated, industrialized, rich and democratic (WEIRD; Azar, 2010) sample lacks reporting of potential ethnic and cultural diversity, as noted in our quality appraisal. Most studies did not locate the researcher theoretically or engage in reflexive practice, and it was not evident if data saturation occurred. Although data may not be rich enough to support external validity, considering the depth of experiences reported from our analysis, we were able to answer our research question. We also identified there is limited reporting of Indigenous and non‐WEIRD nurses experiences. Although our search strategy may have contributed to this, we manually searched common outlets of Indigenous research, including for Indigenous Canadian and American nurses. Future research could explore cultural differences in the nurses' experiences when providing VAD.

Lee et al. (2015) has previously described the lack of procedural clarity and considerable variation in how the seven steps of Noblit and Hare (1988) are undertaken and that different researchers interpreted the methods in unique ways while asserting they were aligned with the method. Our experience was similar, and to overcome this, we engaged in regular discussions to navigate the methods and referred to previous publications. The authors hoped that the inclusion of direct quotations of nurse participants from the primary studies (e.g. Jarden et al., 2021) would reduce bias from doctors and other health professionals' narratives being included in the analysis.

## 8 CONCLUSION

### 8.1 Contribution

This is the first qualitative meta‐synthesis using the ENTREQ reporting guidelines to report nurses' experiences when involved in the care of patients seeking VAD. Nurses contrasted their past experiences using palliative sedation with the ‘beauty’ of a death using VAD where patient autonomy was facilitated, and there was clarity in procedure. We learned that there is significant emotional complexity of working in this area that is partially mitigated by the experience of being within a team, but the nurse carries this weighty load. Despite this weight, nurses felt their experiences were meaningful for themselves and those they cared for, and they felt changed by engaging in VAD. Nurses found that the structures, procedures and policies provided a sense of a shield to support them from some of the perceived negative consequences of engaging in this work.

Nurses gave emotional and personal descriptions of their experiences but their personal reflections on these and how they dealt with and processed these complex emotions were not reported in previous research. We know *what* some of the ethical issues are but less is known about *how* nurses navigate their emotional responses to these within their workplace. Future research to understand nurses' emotional experiences may facilitate workplaces to provide structures and processes to support nurse well‐being.

The present research highlighted the necessity for nurses to feel prepared both emotionally and procedurally for engaging in VAD. This study may inform legislation and policy, as it includes direct experiences of nurses' working with VAD. The need for policy as guidance alongside team support was strongly emphasized by these participants. Strengthening nurses' communication skills with respect to recognizing and expressing emotions, including their triggers (e.g. ethical dilemmas) individually and within a team context, was an important aspect for nurses who had good experiences with VAD.

## AUTHOR CONTRIBUTIONS

All authors have agreed on the final version and meet at least one of the following criteria (recommended by the ICMJE*): (1) Substantial contributions to conception and design, acquisition of data, or analysis and interpretation of data; (2) Drafting the article or revising it critically for important intellectual content.

## FUNDING INFORMATION

This research received no specific grant from any funding agency in the public, commercial or not‐for‐profit sectors.

## CONFLICT OF INTEREST

No conflict of interest has been declared by the author(s).

### PEER REVIEW

The peer review history for this article is available at https://publons.com/publon/10.1111/jan.15324.

## ETHICS STATEMENT

Not sought.

## PROSPERO ID

CRD42021276819.

## Supporting information


Appendix S1

## Data Availability

The data that support the findings of this study are available from the corresponding author, [MS], on reasonable request.
