# Supplementary material for: Nurses' experiences of supporting patients requesting voluntary assisted dying: A qualitative meta‐synthesis
Source: J Adv Nurs. 2022 Jun 24;78(10):3101–15. doi: 10.1111/jan.15324 (PMC9546017; doi:10.1111/jan.15324)
Supplement: Supplementary file 1 — Appendix S1 [file JAN-78-3101-s001.docx]

**Supplementary information**

Search strategy

The search of electronic databases included Cumulative Index to Nursing and Allied Health Literature (CINAHL), Emcare, Medline, Scopus, and PsycInfo. Two reviewers independently screened all titles, abstracts and fulltexts for inclusion based on the specified inclusion and exclusion criteria. The search strategy for Scopus is provided in XXX. The search terms and associated index terms were: “nurs*, “nurs* role”” AND ”assisted dying”, “assisted suicide”, “euthanasia”, “assisted death”, AND , “experience*”, “psycholog*”. MeSH terms for Medline: “Nurses”; “Ethics, nurses”; “Euthanasia”, “Euthanasia, Active”, “suicide, assisted”, “euthanasia, active, voluntary” ; “psychology” NOT “euthanasia, passive”.

CINHAL and Medline

| B ( nurse or nurses or nursing ) AND AB ( euthanasia or assisted suicide or right to die or physician assisted suicide or death with dignity or assisted dying or assisted death) | **Limiters** - English Language; Peer Reviewed; Human; Research Article  **Expanders** - Apply related words; Apply equivalent subjects  **Search modes** - Boolean/Phrase |
| --- | --- |
